# Molecular identification of *Trypanosoma brucei gambiense* in naturally infected pigs, dogs and small ruminants confirms domestic animals as potential reservoirs for sleeping sickness in Chad

**DOI:** 10.1051/parasite/2020061

**Published:** 2020-11-18

**Authors:** Joël Vourchakbé, Zebaze Arnol Auvaker Tiofack, Tagueu Sartrien Kante, Mbida Mpoame, Gustave Simo

**Affiliations:** 1 Molecular Parasitology and Entomology Unit, Department of Biochemistry, Faculty of Science, University of Dschang PO Box 67 Dschang Cameroon; 2 Department of Chemistry-Biology-Geology, Faculty of Science and Technology, University of Doba PO Box 03 Doba Chad; 3 Laboratory of Applied Biology and Ecology (LABEA), Department of Animal Biology, Faculty of Science, University of Dschang PO Box 067 Dschang Cameroon

**Keywords:** Animal reservoir, *Trypanosoma brucei gambiense*, Sleeping sickness, Domestic animals

## Abstract

Human African trypanosomiasis (HAT) has been targeted for zero transmission to humans by 2030. Animal reservoirs of *gambiense*-HAT could jeopardize these elimination goals. This study was undertaken to identify potential host reservoirs for *Trypanosoma brucei gambiense* by detecting its natural infections in domestic animals of Chadian HAT foci. Blood samples were collected from 267 goats, 181 sheep, 154 dogs, and 67 pigs. Rapid diagnostic test (RDT) and capillary tube centrifugation (CTC) were performed to search for trypanosomes. DNA was extracted from the buffy coat, and trypanosomes of the subgenus *Trypanozoon* as well as *T. b. gambiense* were identified by PCR. Of 669 blood samples, 19.4% were positive by RDT and 9.0% by CTC. PCR revealed 150 animals (22.4%) with trypanosomes belonging to *Trypanozoon*, including 18 (12%) *T. b. gambiense*. This trypanosome was found in all investigated animal species and all HAT foci. Between animal species or villages, no significant differences were observed in the number of animals harboring *T. b. gambiense* DNA. Pigs, dogs, sheep and goats appeared to be potential reservoir hosts for *T. b. gambiense* in Chad. The identification of *T. b. gambiense* in all animal species of all HAT foci suggests that these animals should be considered when designing new control strategies for sustainable elimination of HAT. Investigations aiming to decrypt their specific role in each epidemiological setting are important to achieve zero transmission of HAT.

## Abstract (fr)

La trypanosomiase humaine africaine (THA) a été ciblée pour une interruption de sa transmission en 2030. Le réservoir animal de la THA à *Trypanosoma brucei gambiense* pourrait compromettre ces objectifs d’élimination. Cette étude a été entreprise pour identifier des potentiels hôtes réservoirs de *Trypanosoma brucei gambiense* en détectant ses infections naturelles chez des animaux domestiques des foyers tchadiens de la THA. Des échantillons de sang ont été prélevés chez 267 chèvres, 181 moutons, 154 chiens et 67 porcs. Le test de diagnostic rapide (TDR) et la centrifugation en tube capillaire (CTC) ont été utilisés pour mettre en évidence les trypanosomes. L’ADN a été extrait des couches leucocytaires et les trypanosomes du sous-genre *Trypanozoon* ainsi que *T. b. gambiense* ont été identifiés par PCR. Sur les 669 échantillons de sang analysés, 19,4& % étaient positifs au TDR et 9,0& % à la CTC. La PCR a révélé 150 (22,4& %) animaux avec des trypanosomes du sous-genre *Trypanozoon* donc 18 (12& %) portant l’ADN de *T. b. gambiense*. Ce dernier a été identifié chez toutes les espèces animales de tous les foyers de la THA. Entre les espèces animales ou les villages, aucune différence significative n’a été observée entre le nombre d’animaux ayant l’ADN de *T. b. gambiense*. Les porcs, les chiens, les moutons et les chèvres sont apparus comme des hôtes réservoirs potentiels de *T. b. gambiense* au Tchad. L’identification de *T. b. gambiense*, chez toutes les espèces animales étudiées dans tous les foyers, suggère de considérer ces animaux dans la conception des nouvelles stratégies de lutte visant une élimination durable de la THA. Des investigations visant à décrypter leur rôle spécifique dans chaque contexte épidémiologique sont nécessaires pour parvenir à une transmission nulle de la THA.

## Introduction

Human African trypanosomiasis (HAT) also known as sleeping sickness is a neglected tropical disease that affects mostly rural populations of sub-Saharan African countries. Transmitted by an arthropod vector of the genus *Glossina*, this infectious disease is caused by protozoan parasites of the *Trypanosoma brucei* species complex which include *Trypanosoma brucei brucei*, *Trypanosoma brucei gambiense*, and *Trypanosoma brucei rhodesiense*. While *T. b. brucei* infects domestic and wild animals and induces animal African trypanosomiasis, it is non-infective to humans. The other two subspecies are infective to humans and cause HAT. *Trypanosoma b. gambiense* is responsible for the chronic or *gambiense* form of HAT that occurs in West and Central Africa. This form accounts for about 98% of all current HAT diagnosed cases [59]. The acute form of HAT accounts for the remaining 2% of diagnosed cases. Found in Eastern and Southern Africa, this acute form is caused by *T. b. rhodesiense* [59]. During the last few decades, control efforts based on case detection and treatment, sometimes coupled with vector control, made it possible to considerably reduce the incidence of HAT and brought this disease under control with fewer than 2000 cases reported in 2017 [59]. With this success, *gambiense*-HAT has been included in the World Health Organization (WHO) road map that targets its sustainable elimination or interruption of transmission (zero transmission) to humans for 2030 [6, 14, 59]. Achieving these goals requires strengthening of current control activities and identifying components that could jeopardize this sustainable elimination, and that have not been considered in the design of current control measures. It is in this framework that concerns about the epidemiological importance of animal reservoirs have been raised for several decades [11, 36, 57], and recently during the third WHO stakeholders meeting on *gambiense*-HAT elimination that took place in Geneva in April 2018 [59]. Understanding the significance of animal reservoirs in the maintenance of the transmission and re-emergence of *gambiense*-HAT therefore requires further attention.

*Gambiense*-HAT has generally been considered an anthroponotic disease and consequently, its control program was mainly focused on stopping its transmission by treating human cases and eliminating the tsetse vector [6]. However, animal reservoirs have been reported to be one of the epidemiological components that could play an important role in the endemic nature of HAT, and also for its resurgence in the historic foci of West and Central Africa [6, 41]. Previous investigations revealed several domestic and wild animal species as potential hosts of *T. b. gambiense* in West and Central African HAT foci [8, 9, 16, 17, 28, 40–42, 44, 50, 51]. For instance, natural infections of *T. b. gambiense* have been reported in sheep and goats in Cameroon, Equatorial Guinea and Congo [8, 41, 44, 50], in pigs in Cameroon and Liberia [16, 42, 51], and in dogs in Liberia and Nigeria [16, 56]. Besides these domestic species, several wild animal species have been also reported with natural infections of *T. b. gambiense* in Central African HAT foci [10, 17, 40]. In addition to these natural infections, experimental investigations have demonstrated the infectivity and transmissibility of *T. b. gambiense* to several of these animal species. All these data provide evidence of the presence of an animal reservoir that may threaten *gambiense*-HAT elimination. Although the epidemiological role of animals found with *T. b. gambiense* infections has not been deeply investigated in different epidemiological settings, these animals could act as reservoir hosts of *T. b. gambiense* and could maintain and/or ensure its transmission. However, the variability of the fauna composition and the nutritional behavior of tsetse flies in different HAT foci suggest that the epidemiological significance of any animal reservoir may vary according to the specific ecosystems of each HAT focus. Understanding the real epidemiological situation of the disease in each focus is becoming of great importance, especially in some HAT foci of West and Central Africa where elimination could be foreseen.

Chad is among the countries reporting 10 to 100 new cases per year. In Chadian HAT foci, a significant decrease was observed with a 78% reduction in reported cases between 2016 and 2018 [15]. The maintenance of an adequate level of control activities with the implementation of a vector control program in 2014 has greatly reduced the tsetse population in some HAT foci like that of Mandoul [27]. Moreover, only 12 new HAT cases were reported in Chadian HAT foci in 2018 [15]. Although Chad is not yet eligible for validation of *gambiense* HAT elimination as a public health problem at the national level, the low number of new HAT cases highlights progress towards the elimination target. In this context, factors such as animal reservoirs, reported as one of the components that could jeopardize this elimination, must be investigated to identify potential vertebrate hosts for *T. b. gambiense*. In Chadian HAT foci where no investigation on animal reservoirs of *gambiense*-HAT has been undertaken, the epidemiological role of different animal species in the transmission and maintenance of *T. b. gambiense* remains unknown.

The present study aims to generate data on animal reservoirs of *gambiense*-HAT by identifying natural infections of *T. b. gambiense* in domestic animals of HAT foci of Chad.

## Materials and methods

### Ethical considerations

This study was approved by the Bioethics Committee of the Ministry of High Education, Research and Innovation of Chad by decree number 462/PR/PM/MESRI/SG/CNBT/2017. In addition to this approval, the review board of the molecular parasitology and entomology unit of the Department of Biochemistry of the Faculty of Science of the University of Dschang gave its agreement. The local administration, as well as the religious and traditional authorities of each HAT focus also gave their approval after detailed explanation of the objectives of the study. Verbal consent was obtained from animal owners after the aim of the study had been explained to each of them.

### Sampling sites

This cross-sectional study was performed from February 2018 to June 2019 in three HAT foci of the extreme south of Chad. These foci include the Mandoul, Maro and Moissala HAT foci [58].

- The HAT focus of Mandoul (8°6′57″& N; 17°06′58″& E) is located 50& km from Doba, which is the capital of Logone Oriental region. At the borders of Cameroon and the Central African Republic, this HAT focus belongs to areas showing low and very low risk of *T. b. gambiense* infections [15, 46]. It covers 41 villages with an estimated population of 13,799 inhabitants. Its temperature varies from 22 to 38& °C, and the average annual rainfall is 1000& mm [26]. The landscape is dominated by forest galleries and wooded savannahs. In this HAT focus, domestic animals were sampled in 41 villages (Table S1).
- The HAT focus of Maro (8°28′33″& N; 18°46′10″& E) is located 55& km from Sarh, the capital of the Moyen Chari region. It is at the border of the Central African Republic and covers about 33 villages for an estimated population of 8526 inhabitants. It belongs to HAT foci showing moderate risk of *T. b. gambiense* infections [15]. In this focus, the temperature varies from 25 to 38& °C and the precipitation from 800& mm to 1300& mm. The vegetation is formed by savannah and clear forests dotted with trees. For this study, animals were sampled in 31 villages.
- The HAT focus of Moïssala (8°20′25″& N; 17°45′58″& E) is part of the great historical HAT focus of Middle Chari [3]. It extends along the network dominated by the Nana-Barya river, between the Bahr Sara (Ouham) and Chari rivers. Located in the South of Koumra, the Capital of the Mandoul region, about 400& km from the Central Africa Republic border, the Moissala focus belongs to HAT foci showing moderate risk of *T. b. gambiense* infections [14, 15]. Its population is estimated to be 12,234 inhabitants distributed in 41 villages. In this HAT focus, the temperature varies from 24 and 38& °C with an average annual rainfall of about 1100& mm. The vegetation is dominated by forest galleries. Domestic animals of 42 villages were sampled.

Inhabitants of these three Chadian HAT foci are mainly traditional smallholder farmers. The main crops are cotton, millet and sesame. Inhabitants also practice fishing, gathering, hunting, extensive breeding of cattle, and small-scale breeding of sheep, goats, pigs, and equines that are generally used for transport and traction. These different animal species move in and around biotopes that are favorable to tsetse flies and, consequently, are exposed to tsetse bites, like humans.

### Sample collection

Domestic animals including pigs, dogs, sheep and goats were sampled during three field surveys performed in villages of each of the three HAT foci of Chad. The sampling was done in all villages where at least one HAT case was reported during the last decade, as well as in some neighboring villages. These villages were selected on the basis of (i) their proximities with villages where an HAT case has been reported; (ii) the presence of different domestic animal species; and (iii) the presence of favorable biotopes for tsetse flies and trypanosome transmission. The first survey was performed from 7 to 27 March 2018 in the HAT focus of Maro, the second from 2 April to 12 May 2018 in the Mandoul HAT focus, and the last survey from 25 May to 14 June 2019 in the Moissala HAT focus. Before each survey, the objective of the study was explained for the second time to inhabitants and local authorities of each HAT focus. One day before each sampling, inhabitants of each village were asked to restrain and/or tie their animals around their houses. In each village, only animals that may have been exposed to trypanosome infections or tsetse bites by spending at least 3 months in the study zone were selected. For animal owners having fewer than three animals, all their animals were sampled, while for those having more than three animals satisfying the previous criterion, only three animals were randomly selected. With the cooperation of owners, about 5& mL of blood were collected from each domestic animal. Each collection was done into EDTA coated tubes. The blood collection was performed from the jugular vein in goats and sheep while in pigs and dogs, it was performed from the sub-clavicular and cephalic vein, respectively. In some dogs, blood samples were not collected due to their aggressiveness. Each blood tube was labelled and carefully packed. All dogs sampled in the three HAT foci were of local breed, originating from a mixture of different breeds. The pigs were Iberian pigs, “Landschwein pigs” and a mixture of different breeds. The sheep were “south sheep” made up of sheep of “Kirdi” or “Kirdimi” type and sheep of Mayo-Kebbi [12]. The goats were also “south goat” or “Kirdimi goat” or Guinean goats with djallonke and dwarf kirdi African goat breeds [12]. The goats and sheep breed sampled in this study have been reported to be able to control trypanosome infections [39].

### Immunologic test and parasitological examination

The immunologic test or *gambiense*-HAT rapid diagnostic test (RDT) was performed to identify animals that would have been in contact with *T. b. gambiense*. The RDT named SD BIOLINE HAT was the test used in this study. Developed using native variable surface glycoproteins (VSGs) (Nat-LiTat 1.3 and Nat-LiTat 1.5) obtained from the Institute of Tropical Medicine (ITM) of Belgium [29], this test detects anti-VSG LiTat 1.3 and anti-VSG LiTat 1.5 antibodies [2, 21, 55]. This RDT was performed as described by Matovu et al. [29].

The capillary tube centrifugation test (CTC) was performed on each blood sample to search for trypanosomes. This was performed as described by Woo [60]. All animals found with trypanosome infections through the CTC were treated. Infected goats, sheep and dogs were treated with 0.5& mg of Trypamidium per 1& kg body weight, while pigs were treated with 0.5& mg of Quinapyramine per 1& kg body weight following local veterinarian’s advice.

The blood samples remaining in EDTA coated tubes were centrifuged at 13,000& rpm for 5& min. After centrifugation, the buffy coat was removed from each tube and transferred to microtubes of 1.5& mL that were stored into an electric cooler. These buffy coat samples were transported to the molecular parasitology and entomology unit of the Faculty of Science of the University of Dschang in Cameroon. They were transported in an electric cooler and once at the laboratory, they were subsequently stored at −20& °C until DNA extraction for molecular analyses.

### DNA extraction

DNA was extracted from each buffy coat using the cethyl trimethyl ammonium bromide (CTAB) method. In each microtube of 2& mL, 500& μL of buffy coat were mixed with 1& mL of nuclease-free water. After vigorous homogenization of each microtube, the mixture was centrifuged at 11,000& rpm for 15& min. The supernatant was removed and 600& μL of CTAB buffer (CTAB at 5%; 1& M Tris, pH 8; 0.5& M EDTA, pH 8; 5& M NaCl) were added to the pellet that was re-suspended and incubated in a water bath at 60& °C for 30& min. Thereafter, 600& μL of chloroform/isoamyl alcohol (24/1) mixture were added to the resulting pellet of each microtube. This mixture was slowly homogenized for 15& min and then centrifuged at 13,000& rpm for 15& min. The upper aqueous phase was removed and transferred to another microtube of 1.5& mL. DNA was precipitated by addition of 600& μL of isopropanol. After gentle homogenization of each microtube for 5& min and its incubation overnight at −20& °C, each microtube was centrifuged at 13,000& rpm for 15& min. DNA pellet was subsequently washed twice with cold 70% ethanol and dried overnight at room temperature. The resulting DNA pellet was re-suspended in 50& μL of sterile nuclease free water and subsequently stored at −20& °C until use.

### Molecular identification of trypanosomes of the subgenus *Trypanozoon*

Molecular identification was done as described by Simo et al. [51] using TBR-1/2 primers that are specific to trypanosomes of the subgenus *Trypanozoon* [38]. PCR reactions were performed in a final volume of 25& μL containing 5& mL of DNA extracts as template, 1× PCR buffer (10& mM Tris-HCl (pH 9), 50& mM KCl), 2& mM MgCl_2_, 20 picomoles of each primer (TBR-1: 5′–GAATATTAAACAATGCGCAG–3′; TBR-2: 5′–CCATTTATTAGCTTTGTTGC–3′), 100& mM of each dNTP and 1 unit of Taq DNA polymerase. The amplification program contained one cycle of denaturation at 94& °C for 5& min followed by 40 amplification cycles. Each of these cycles was made up of a denaturation step at 94& °C for 30& s, an annealing step at 55& °C for 30& s, and an extension step at 72& °C for 1& min. This was followed by a final extension at 72& °C for 10& min.

The amplified products were separated by electrophoresis on 1.5% agarose gel that was subsequently stained with ethidium bromide and visualized under UV light. A sample was considered positive for trypanosomes of the subgenus *Trypanozoon* if a DNA fragment of about 177& bp was observed after amplification with TBR-1/2 primers. In addition to this DNA fragment of 177& bp, other DNA fragments can be observed because the target fragment is a satellite DNA sequence with repetitions of different sizes. For subsequent analyses, all samples that were positive for trypanosomes of the subgenus *Trypanozoon* were selected.

### Molecular identification of *T. b. gambiense*

Searching for *T. b. gambiense* was performed on all samples that have shown a DNA fragment of about 177& bp corresponding to the size expected for trypanosomes of the subgenus *Trypanozoon* (*T. b. brucei, T. evansi, T. b. gambiense, T. b. rhodesiense* or *T. equiperdum*). The molecular identification of *T. b. gambiense* was done using two PCR rounds as described by Cordon-Obras et al. [8]. During this identification, two pairs of specific primers for *T. b. gambiense* were used: TgSGP-1/2 (TgSGP-1: 5′–GCT GCT GTG TTC GGA GAG C–3′; TgSGP-2: 5′–GCC ATC GTG CTT GCC GCT C–3′) described by Radwanska et al. [47] and TgsGP-a/as (TgsGP-s: 5′–TCA GAC AGG GCT GTA ATA GCA AGC-3′; TgsGP-as: 5′–GGG CTC CTG CCT CAA TTG CTG CA–3′) designed by Morrison et al. [37].

The first PCR round was carried out in a total volume of 25& μL containing 1× PCR buffer (Tris – 10& mM HCl (pH 9.0), 50& mM KCl, 3& mM MgCl_2_), 15 picomoles of each primer (TgSGP-1/2), 100& mM of each dNTP, one unit of Taq DNA polymerase, 5& μL of DNA extract and 14& μL of sterile water. The amplification program contained an initial denaturation step at 95& °C for 3& min. This was followed by 45 cycles made up of a denaturation step at 95& °C for 30& s, an annealing step at 63& °C for 1& min and an elongation step at 72& °C for 1& min. A final elongation was done at 72& °C for 5& min. Amplicons of the first PCR were diluted 10 times and 5& μL of each dilution were subsequently used as DNA template for the second PCR in which TgsGP-s/as primers were used. In this nested PCR, only 25 amplification cycles were performed in the same conditions as for the first PCR.

Amplicons from the second PCR were resolved by electrophoresis on 2% agarose gel containing ethidium bromide (0.3& μg/mL). DNA bands were subsequently visualized and then photographed under UV light. All samples in which a DNA fragment of about 270& bp was revealed after PCR and electrophoresis were considered positive for *T. b. gambiense*.

### Data analyses

The infection rates of trypanosomes of the subgenus *Trypanozoon* and those of *T. b. gambiense* were compared between animal species and HAT foci. These comparisons were performed using XLSTAT 2016 software. The chi-squared test (*χ*^2^) was used to compare the infection rates between animal species and different HAT foci. The difference was considered significant if the *p*-value was below 0.05.

## Results

The 669 domestic animals analyzed in this study were sampled in 113 villages: 41, 31 and 41 villages of the Mandoul, the Maro and the Moissala HAT foci, respectively (Table S1). These 669 animals include 267 goats (39.9%), 181 sheep (27.1%), 154 dogs (23.0%), and 67 pigs (10.0%) (Table 1). Of these 669 animals, 268 (40.1%), 232 (34.7%) and 169 (25.3%) were sampled in the HAT foci of Mandoul, Maro and Moissala, respectively (Table 2). Details regarding the distribution of domestic animals according to villages of each HAT focus are reported in Table S1.

**Table 1:** Trypanosome infections according to animal species.

Animal species	NE	RDT+ (%)	T+ (%)	PCR results	
				TB+ (%)	TBG+ (%)
Goat	267	53 (19.8)	28 (10.4)	66 (24.7)	8 (3.0)
Sheep	181	37 (20.4)	13 (7.2)	44 (24.3)	5 (2.8)
Dog	154	29 (18.8)	13 (8.4)	32 (20.8)	4 (2.6)
Pig	67	11 (16.4)	6 (8.9)	8 (11.9)	1 (1.5)
Total	669	130 (19.4)	60 (9.0)	150 (22.4)	18 (2.7)
*χ*^2^	–	2.09	1.51	5.65	1.03
*p*-value	–	0.91	0.68	0.13	0.96

NE: Number of animals examined; RDT: Rapid diagnosis test; T+: trypanosome infections revealed by capillary tube centrifugation; TB+: trypanosomes of the subgenus *Trypanozoon*: TBG: *Trypanosoma brucei gambiense*.

**Table 2:** Trypanosome infections according to HAT foci.

HAT foci	Villages	NE	RDT+ (%)	T+ (%)	PCR results	
					TB+ (%)	TBG+ (%)
Mandoul	41	268	51 (19.0)	20 (7.5)	43 (16.0)	12 (4.5)
Maro	31	232	44 (19.0)	22 (9.5)	51 (22.0)	4 (1.7)
Moissala	41	169	35 (20.7)	18 (10.7)	56 (33.1)	2 (1.2)
Total	113	669	130 (19.4)	60 (9.0)	150 (22.4)	18 (2.7)
*χ*^2^	–	–	3.23	1.41	17.44	5.39
*p*-value	–	–	0.52	0.50	0.0001	0.07

HAT: Human African Trypanosomiasis; NE: Number of animals examined; RDT: Rapid diagnosis test; T+: trypanosome infections revealed by capillary tube centrifugation; TB+: trypanosomes of the subgenus *Trypanozoon*: TBG: *Trypanosoma brucei gambiense*.

### Serology

The RDT was positive in 130 animals (19.4%): 53 goats (19.8%), 37 sheep (20.4%), 29 dogs (18.8%), and 11 pigs (16.4%). The positivity rate of RDT varied within and between HAT foci (Table S1). Comparing the positivity rates of RDT, no statistically significant difference (*χ*^2^& =& 2.09; *p*& =& 0.91) was observed between different animal species (Table 1). Although the positivity rates in RDT varied slightly between HAT foci, no significant difference (*χ*^2^& =& 3.23*; p*& =& 0.52) was observed (Table 2).

### Parasitology

The CTC test revealed 60 (9.0%) animals with trypanosome infections. At this stage, the specific identification of trypanosomes was not possible. All four domestic animal species were found with trypanosome infections at all the study sites (Tables 1 and 2). The proportion of infected animals was highest in goats (10.4%) followed by pigs (8.9%). The lowest infection rate of 7.2% (13/181) was observed in sheep (Table 1). However, these differences in infection rates between animal species were not statistically significant (*χ*^2^& =& 1.51; *p*& =& 0.68) (Table 1).

Of the 60 animals in which trypanosome infections were revealed by CTC, 22 (9.5%), 20 (7.5%), and 18 (10.7%) were from the HAT foci of Maro, Mandoul and Moissala, respectively (Table 2). Between these HAT foci, no significant difference (*χ*^2^& =& 1.14; *p*& =& 0.50) was observed in the trypanosome infection rates revealed by CTC (Table 2).

### Molecular analyses

#### Molecular identification of trypanosomes of the subgenus *Trypanozoon*

From the 669 animals analyzed in this study, the specific PCR targeting a multi-copy 177& bp repeat sequence of trypanosomes of the subgenus *Trypanozoon* revealed this sequence in 150 animals (22.4%). Sixty six goats (24.6%), 44 sheep (24.30%), 32 dogs (20.8%), and 8 pigs (11.9%) were tested positive for trypanosomes of the subgenus *Trypanozoon*. Despite some small differences observed in these trypanosome infection rates, no significant difference (*χ*^2^& =& 5.65; *p*& =& 0.13) was found between animal species.

Of the 150 animals found with trypanosomes of the subgenus *Trypanozoon,* 56 (33.1%) were from the Moissala HAT focus, 51 (22.0%) from Maro, and 43 (16.0%) from Mandoul. The highest infection rate of 33.1% (56/169) was observed in animals of the HAT focus of Moissala, while the lowest rate of 16.0% was observed in those of the Mandoul HAT focus (Table 2). Infections due to trypanosomes of the subgenus *Trypanozoon* were widely distributed in animals of different villages of the three HAT foci (Figs. 2–4). The difference between the trypanosome infection rates revealed by PCR in the three HAT foci was statistically significant (*χ*^2^& =& *1*7.44; *p*& =& 0.0001) (Table 2).

Fifty seven out of the 150 animals (38.0%) that were positive for PCR targeting trypanosomes of the subgenus *Trypanozoon* were tested positive for trypanosome infection by CTC. Three out of the 60 animals that were positive in CTC were tested negative in the PCR targeting trypanosomes of the subgenus *Trypanozoon*.

#### Molecular identification of *T. b. gambiense* infections

**Figure 1:**
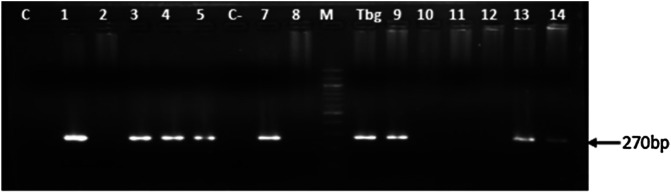
Photo of agarose gel showing specific DNA fragments of *T. b. gambiense* that were amplified from domestic animals. C−: Negative control, Tbg: Positive control made of purified DNA from *T. b. gambiense* human isolate; M: Molecular marker (100& bp ladder); 1, 3, 4, 5, 7, 9, 13 and 14: samples with *T. b. gambiense* infections; 2, 8, 10, 11 and 12: samples without *T. b. gambiense* infection, but harboring other trypanosomes of the subgenus *Trypanozoon*.

Of the 150 animals tested positive for trypanosomes of the subgenus *Trypanozoon* by the first PCR, 18 (12.0%) of them were found with *T. b. gambiense* infections as shown by the amplification of specific DNA fragments of 270& bp of this trypanosome subspecies (Fig. 1). All four animal species investigated in this study were found with *T. b. gambiense* infections (Table 1). Of the 18 animals found with *T. b. gambiense* infections, 8 (44.4%), 5 (27.8%), 4 (22.2%) and 1 (5.6%) were from goats, sheep, dogs and pigs, respectively. The overall *T. b. gambiense* infection rate was 2.7% (18/669). The highest infection rate of 3.0% (8/268) was found in goats, followed by sheep (2.8%), dogs (2.6%), and pigs (1.5%) (Table 1). Comparing the infection rates of *T. b. gambiense*, no significant difference (*χ*^2^& =& 1.03; *p*& =& 0.96) was observed between animal species (Table 1). Of the 60 animals revealed with trypanosome infections by CTC, the PCR targeting *T. b. gambiense* detected this trypanosome subspecies in 10 (16.7%) of them. Eight animals with DNA of *T. b. gambiense* and selected by PCR targeting trypanosomes of the subgenus *Trypanozoon* were negative to the parasitological test (CTC).

**Figure 2:**
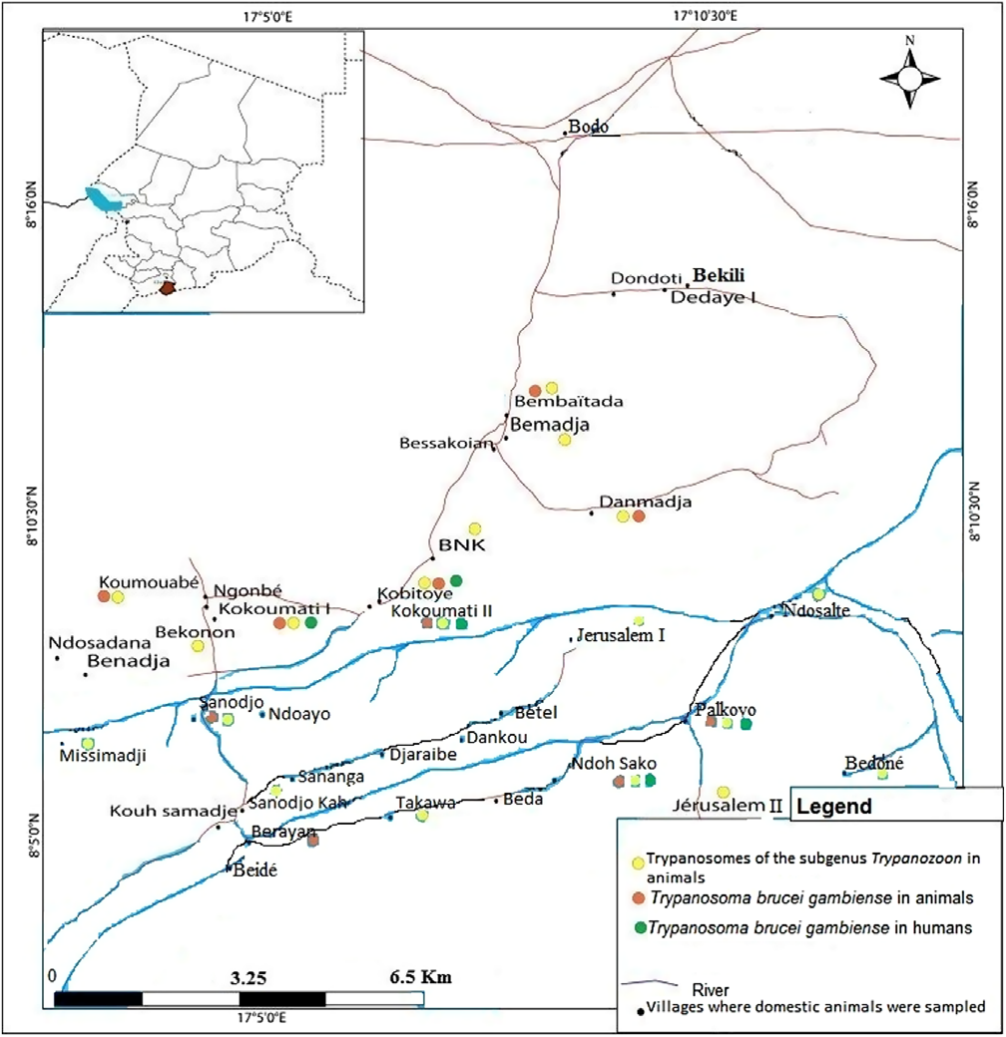
Distribution of trypanosome infections in humans and animals of the sleeping sickness focus of Mandoul. Green dots represent villages where HAT cases have been reported by the national sleeping sickness control program of Chad; yellow dots represent villages where animals were found with trypanosomes of the subgenus *Trypanozoon*; orange dots represent villages where animals were found with *T. b. gambiense* infections.

**Figure 3:**
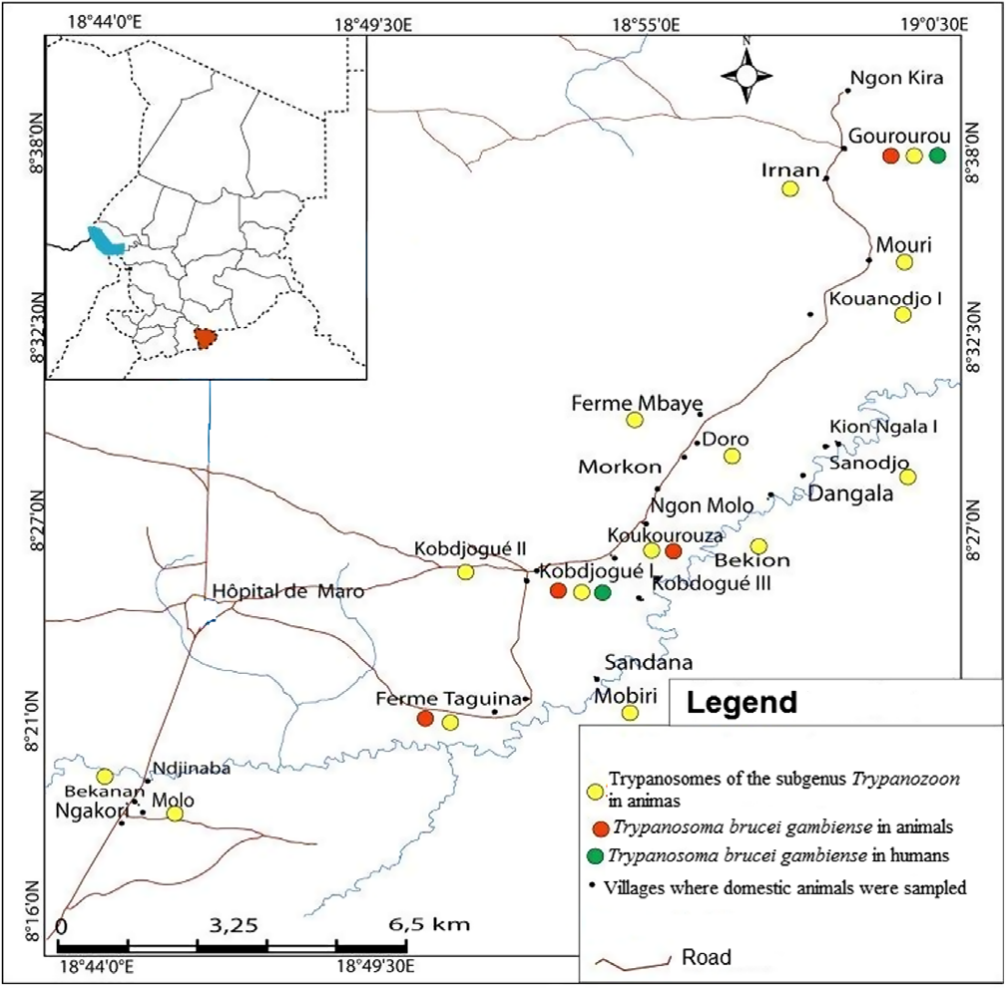
Distribution of trypanosome infections in humans and animals of the sleeping sickness focus of Maro. Green dots represent villages where HAT cases have been reported by the national sleeping sickness control program of Chad; yellow dots represent villages where animals were found with trypanosomes of the subgenus *Trypanozoon*; orange dots represent villages where animals were found with *T. b. gambiense* infections.

**Figure 4:**
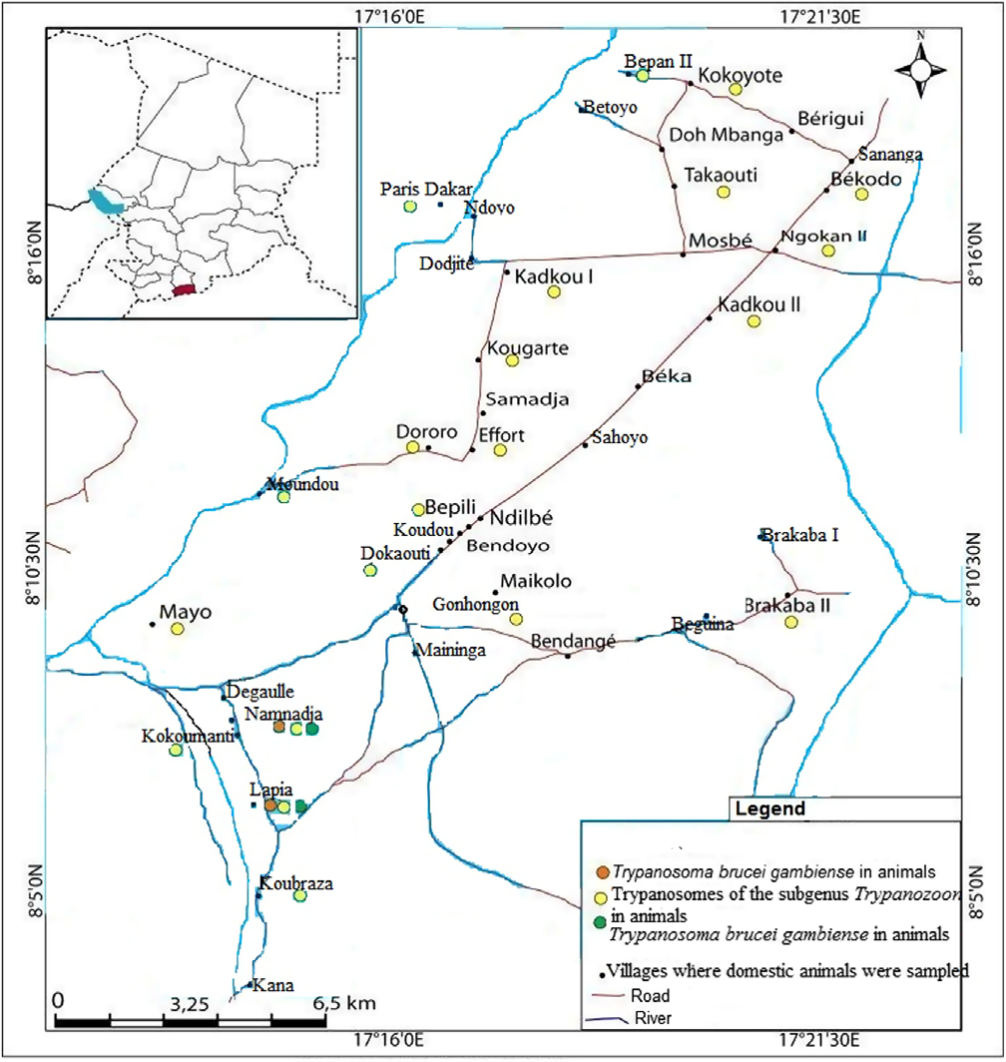
Distribution of trypanosome infections in humans and animals of the sleeping sickness focus of Moissala. Green dots represent villages where HAT cases have been reported by the national sleeping sickness control program of Chad; yellow dots represent villages where animals were found with trypanosomes of the subgenus *Trypanozoon*; orange dots represent villages where animals were found with *T. b. gambiense* infections.

Animals with *T. b. gambiense* infections were identified in each HAT focus investigated in this study. Of the 18 animals tested positive for *T. b. gambiense* infections, 12 (66.7%) were from the HAT focus of Mandoul (Fig. 2), while 4 (22.2%) and 2 (11.1%) were from the Maro (Fig. 3) and Moissala (Fig. 4) HAT foci, respectively. In all villages that have reported *T. b. gambiense* infections in humans, domestic animals were also found with human infective trypanosome (Figs. 2–4). However, in two and five villages of the HAT foci of Maro and Mandoul, respectively where no HAT case was reported, animals were found with *T. b. gambiense* infections. The overall highest infection rate of *T. b. gambiense* of 4.5% was reported in animals of the HAT focus of Mandoul. This was followed by 1.7% and 1.2% in the HAT foci of Maro and Moissala, respectively. Between these HAT foci, no significant difference (*χ*^2^& =& 5.39; *p*& =& 0.07) was observed in the overall infection rates of *T. b. gambiense* (Table 2).

### Distribution of trypanosome infections between villages and foci

Amongst the 113 villages where the sampling was undertaken, animals were tested positive for *T. b. gambiense* in 16 of them (11.3%): 2 villages of the Moissala HAT focus, 4 of Maro, and 10 of Mandoul. Two animals from Kokoumati I and two others from Kobitoye, all of the Mandoul HAT focus, were positive for *T. b. gambiense*. Trypanosomes of the subgenus *Trypanozoon* were found in animals from 56 villages: 20, 15 and 21 villages of the HAT foci of Mandoul, Maro and Moissala, respectively (Table S1). Of these 56 villages, animals with *T. b. gambiense* infections were reported in 16 of them: 10 villages of the Mandoul HAT focus, 4 of Maro, and 2 of Moissala (Table S1). Fifty seven villages including 21, 16 and 20 villages of the HAT foci of Mandoul, Maro and Moissala, respectively reported no animal with trypanosomes of the subgenus *Trypanozoon*. The difference in the number of villages reporting animals with trypanosomes of the subgenus *Trypanozoon* was not statistically significant (*χ*^2^& *=*& 1.66; *p*& =& 0.44) between HAT foci. For villages reporting animals with *T. b. gambiense* infections, a significant difference (*χ*^2^& *=*& 9.75; *p*& =& 0.008) was observed between HAT foci. Between villages with and without HAT cases, there was no significant difference (*χ*^2^& *=& 4.0*; *p*& =& 0.05 for the HAT focus of Moissala; *χ*^2^& *=& 0.0*; *p*& =& *1* for the HAT foci of Mandoual and Maro) in the number of animals that were positive for *T. b. gambiense*. Comparing the number of animals with *T. b. gambiense* infections in villages reporting both HAT cases and positive animals for *T. b. gambiense*, no significant difference (*χ*^2^& *=& 3.0*; *p*& =& 0.22) was observed between HAT foci. For similar comparison between villages without HAT cases, but reporting positive animals for *T. b. gambiense*, a significant difference (*χ*^2^& *=*& 8.143; *p*& =& 0.02) was observed.

Among the 16 villages reporting animals tested positive for *T. b. gambiense*, HAT cases were reported in 9 (56.3%) of them. Of the 104 villages with no HAT case, animals positive for *T. b. gambiense* were reported in 7 (6.7%) of them. No HAT case and no animal positive for *T. b. gambiense* were reported in 97 (85.8%) villages. Moreover, no village reported HAT case and animals that were tested negative for *T. b. gambiense*. Considering villages reporting positive animals for *T. b. gambiense*, the difference between the numbers of villages with (9) and without (7) HAT case was not statistically significant (*χ*^2^& *=*& 0.48; *p*& =& 0.49). For villages reporting no HAT case, a significant difference (*χ*^2^& *=*& 155.026; *p& <*& 0.0001) was observed between the numbers of villages with (7) and without (97) positive animals for *T. b. gambiense*. Between the numbers of villages with (16) and without (97) animals positive for *T. b. gambiense*, a significant difference (*χ*^2^& *=*& 3.84; *p& <*& 0.0001) was also observed.

### Comparison between RDT and PCR targeting *T. b. gambiense* and trypanosomes of the subgenus *Trypanozoon*

RDT targeting antibodies against *T. b. gambiense* and PCR targeting trypanosomes of the subgenus *Trypanozoon* were simultaneously positive and negative for 57 (8.5%) and 446 (66.7%) animals, respectively. Moreover, RDT was positive in 73 animals (10.9%) that were negative by PCR, while trypanosomes of the subgenus *Trypanozoon* were detected by PCR in 93 animals (13.9%) negative to RDT.

For RDT targeting antibodies for *T. b. gambiense* and PCR targeting *T. b. gambiense*, 549 samples (82.1%) revealed concordant results for the two tests, while discordant results were reported in 120 samples (17.9%) (Table 3). Of the 549 samples with concordant results, 14 (2.6%) and 535 (97.4%) were positive and negative for both tests, respectively (Table 3).

**Table 3:** Concordance and discordance between RDT and PCR targeting *Trypanosoma brucei gambiense* according to animal species.

Animal species	Concordance		Discordance	
	RDT+/PCR_TBG+	RDT−/PCR_TBG−	RDT+/PCR_TBG−	RDT−/PCR_TBG+
Goat	6	212	47	2
Sheep	4	143	33	1
Dog	3	124	26	1
Pig	1	56	10	0
Total	14	535	116	4

RDT: rapid diagnostic test; PCR: polymerase chain reaction; TBG: *Trypanosoma brucei gambiense*.

Additional comparisons between RDT and PCR results were undertaken to see whether the performance of these tests could vary according to animal species. The data resulting from these comparisons are summarized in Table 3.

## Discussion

To complete data generated on tsetse and *T. b. gambiense* infections in humans of most *gambiense*-HAT foci of West and Central Africa, natural infections of *T. b. gambiense* were investigated in domestic animals of three HAT foci of Chad. The *gambiense*-HAT RDT used in this study is an immuno-chromatographic test developed for the screening of *gambiense*-HAT. Expected to be positive when the host has been in contact with *T. b. gambiense* [4, 5], results of seroprevalence suggest that 19.4% of animals analyzed would have been in contact with *T. b. gambiense*. However, for the same samples, only 2.7% of animals were found with DNA of *T. b. gambiense*. Results of seroprevalence could be explained by the fact that a positive RDT could indicate current or past infections, while a positive PCR can be interpreted as an active infection although problems of reproducibility of PCR for diagnosis of HAT have been reported by Koffi et al. [24]. We cannot rule out the fact that some positive RDTs could result from cross-reactions with epitopes of other trypanosome species [29]. In fact, sera from animals infected with either *T. congolense*, *T. b. brucei* or *T. evansi* have been shown to cross-react with antigens used in the RDT and the card agglutination test for *gambiense* human African trypanosomiasis [18, 43, 61]. Moreover, sera from both *T. b. gambiense* and *T. b. rhodesiense* patients have induced cross-reactions with a large number of trypanosome antigens including VSG LiTat 1.3 and VSG LiTat 1.5 [1]. The identification of *T. congolense* and *T. vivax* in equines of the same HAT foci [58] and the fact that three animals found with trypanosome infections by CTC, but in which trypanosomes of the subgenus *Trypanozoon* was not detected by PCR, suggest the presence of other trypanosome species in the study area. In addition, the high degree of similarity reported in the genomes of some trypanosomes highlights the probability of cross-reactions between antigens of different trypanosome species [19, 54]. Our results are in agreement with the very few published data reporting the use of RDT on domestic animals [28, 58]. They confirm the low specificity of RDT for the identification of *T. b. gambiense* in animals. This test is therefore not suitable for screening of *T. b. gambiense* in domestic livestock.

The infection rate of 22.4% revealed by PCR targeting trypanosomes of the subgenus *Trypanozoon* is higher than 9% obtained with CTC. Although the molecular tests have high sensitivity and excellent specificity in the detection of various trypanosomes species and subspecies, no single test is able to differentiate unequivocally trypanosomes of the sub genus *Trypanozoon* [7]. The animals found with trypanosomes of the subgenus *Trypanozoon* could be infected by *T. evansi* or *T. equiperdum* or *T. b. brucei* or *T. b. gambiense* or a mixture of some of them. *Trypanosoma b. rhodesiense* is not expected in this study because the Chadian HAT foci are located in a non-endemic area for the acute form of HAT. Remarkably, only 16.7% of animals with trypanosome infections revealed by CTC were found with DNA of *T. b. gambiense*. These animals are probably carriers of significant parasite load that can be detected by parasitological tests. Nevertheless, it important to point out that some of these animals may harbor mixed infections of *T. b. gambiense* with other trypanosome species such as *T. congolense*, *T. vivax* or *T. b. brucei* that could induce significant parasite load [44]. Despite this parasite load, it is not possible to differentiate the infecting trypanosomes species or subspecies, especially *T. b. brucei* from *T. b. gambiense* which are morphologically identical. Animals tested positive for trypanosome infections only by PCR were probably carriers of trypanosomes presenting low parasitemia below the detection threshold of CTC. Finding some animals with *T. b. gambiense* infections that were negative for the parasitological tests is not surprising because experimental studies have shown that such infections in animals are generally characterized by low parasitemia [44].

The identification of trypanosomes of the subgenus *Trypanozoon* in pigs, goats, sheep and dogs of Chadian HAT foci is in agreement with results reported in HAT foci of West and Central Africa [20, 41, 42, 51, 53]. Between animal species, the present study revealed no significant difference in the infection rate of trypanosomes of the subgenus *Trypanozoon*, while significant differences were reported in Cameroonian HAT foci [38, 39]. Such differences could be explained by the livestock breeding system that differs between HAT foci of different countries or the low power of the present study. In Chadian HAT foci, most domestic animals move freely and share the same biotopes, while in Cameroon, pigs are most often kept in pigsties and therefore, are not exposed to the same level of tsetse bites as other animal species. The significant difference observed between HAT foci for the infection rates of trypanosomes of the subgenus *Trypanozoon* is in line with results of other Central African countries and suggests that the transmission patterns could vary according to HAT foci [41].

Previous entomological data reporting *Glossina tachinoides, G. fuscipes fuscipes* and *G. morsitans submorsitans* in HAT foci of Chad [46] suggest that among the 150 animals found with trypanosomes of the subgenus *Trypanozoon*, some may be infected by trypanosomes belonging to the *T. brucei* species complex. This hypothesis is confirmed by the detection of *T. b. gambiense* in animals of the three HAT foci. The identification of natural infections of *T. b. gambiense* in pigs, sheep, goats and dogs confirm results obtained in some West and Central African HAT foci [8, 9, 16, 17, 28, 40–43, 50, 51, 56]. Moreover, experimental studies have demonstrated that all animal species found with *T. b. gambiense* can maintain this parasite for several months with the possibility of completing its cyclical transmission [35]. These animals can therefore be considered potential hosts for *T. b. gambiense* in HAT foci of Chad.

Our results showing no significant difference in the infection rates of *T. b. gambiense* between animal species and HAT foci are not in agreement with those reported in Cameroon [41]. In Cameroonian foci, the infection rates of *T. b. gambiense* varied significantly from zero in dogs to 6.7% in sheep, and from zero in the HAT focus of Doume to 4.8% at Campo [41]. The discrepancy observed suggests that the transmission patterns of *T. b. gambiense* may vary between HAT foci or animal species. This variation could be linked to the fauna composition, the density and tsetse species, the behavior of vertebrate hosts and the nutritional preference of tsetse in each HAT focus or the study design. Located in the southern part of Chad, the three HAT foci have more likely the same fauna composition as well as similar biotopes. Living and moving together in similar environmental conditions, all animal species that were investigated in the present study may be subjected to the same level of tsetse bites. In such a context, the transmission pattern of *T. b. gambiense* could be similar in Chadian HAT foci although additional investigations are required to confirm this hypothesis. In Cameroonian HAT foci, the transmission pattern may vary because the fauna composition and the nutritional preference of tsetse flies vary according HAT foci [13, 40, 41, 52].

The discrepancies observed in the infection rates of *T. b. gambiense* between animal species and HAT foci of Cameroon and Chad could be also explained by the actors involved in the transmission cycle of *T. b. gambiense* in each epidemiological setting. In some villages of Mandoul and Maro HAT foci where infections due to *T. b. gambiense* were reported in animals but not in humans, the transmission cycle may involve tsetse and domestic animals. This type of transmission cycle has already been reported in some Central African HAT foci [8–10, 52]. The transmission cycle could be different in other villages where *T. b. gambiense* were reported in both humans and domestic animals. This cycle may involve humans, animals, and tsetse flies. Such mixed transmission cycles have been reported in Cameroonian HAT foci [52]. Our study shows that investigations on animal reservoirs in different epidemiological settings are of great value for understanding the transmission of *T. b. gambiense*. Our results indicate that reaching the total interruption of HAT transmission by 2030, as indicated in the WHO Road Map, requires an understanding of the frequency at which *T. b. gambiense* could be transmitted from animal reservoirs to humans.

Taking together results of *T. b. gambiense* infections in animals with those deriving from medical surveys performed the same year in humans of the same HAT foci (National sleeping sickness control program, 2018, unpublished data), it appeared that 18 animals were found with *T. b. gambiense* infections and 12 new HAT cases were detected. Although a direct comparison cannot be done between infections in humans and animals due to variations in the population size and host species, the spatial distribution of *T. b. gambiense* infections in humans and animals shows that, in all villages where HAT cases have been reported, domestic animals were found with *T. b. gambiense* infections. The number of villages in which animals were found with *T. b. gambiense* is higher than that reporting such infections in humans. In the Mandoul HAT focus where 12 animals of 10 villages were found with *T. b. gambiense* infections, HAT cases were reported in only five villages. However, in the HAT focus of Moissala, *T. b. gambiense* were reported in humans and animals of the same villages. These results show that the epidemiological situation of HAT and especially the animal reservoir and the transmission of *T. b. gambiense* may vary between HAT foci. The concomitant presence of *T. b. gambiense* infections in animals and humans of the same village highlights a probable link between infections in humans and animals. It suggests a possible transmission of *T. b. gambiense* between these hosts and probably an epidemiological connection between infections of *T. b. gambiense* in humans and animals. Clearly, the domestic animals found with *T. b. gambiense* can move freely in and around villages. Sharing the same environment with humans, tsetse flies can take their blood meals either on humans or domestic animals and therefore, can become infected or ensure the transmission to both humans and animals. For villages reporting *T. b. gambiense* infections in animals and no HAT case in humans, some hypotheses can be formulated to explain these infections. Considering inhabitants’ movement within and between villages surrounding each HAT focus, it is likely that tsetse can become infected by feeding on HAT patients who visit villages where no HAT case has been reported. If *T. b. gambiense* completes its development cycle, infected flies can ensure the transmission to animals during blood meals. Some animals with *T. b. gambiense* infections could acquire these infections in HAT endemic villages during their movements. Although the movement of tsetse flies is generally limited in space, we cannot rule out the fact that infected flies could migrate from HAT endemic villages to those where no HAT cases have been reported.

In both humans and animals, parasite transmission can be performed by the same vector and can occur in the same environment. Control operations could therefore have an impact on the infection rates of *T. b. gambiense* in humans and animals. The medical surveys regularly undertaken in the three HAT foci have continuously reduced the number of human infections. Before and during our sampling period, no control strategy for animal African trypanosomiases was undertaken in these HAT foci. The high infection rate reported in animals could result from infections dating back several months or years because most animals found with *T. b. gambiense* can maintain this parasite for many years [32, 33, 45, 49]. In such infected animals, *T. b. gambiense* conserves its biochemical and human infectivity markers for more than one year, and therefore, could maintain the parasite and ensure its transmission to humans and animals [34]. The cyclical transmission of *T. b. gambiense* that has been demonstrated between tsetse, goats, sheep, pigs, dog and various wild animals is another argument highlighting the epidemiological role of animal reservoirs in the *gambiense*-HAT [15, 25, 30, 31, 48, 57]. In some contexts, animals found with *T. b. gambiense* can maintain these latent infections for several years. In consequence, the persistent of “residual foci” and the sporadic resurgence of some HAT foci may result from the existence of latent infections in domestic or wild animal reservoirs known as source of blood meals for tsetse [11, 22, 23].

In the three HAT foci, sampling was not performed at the same period. This may have potential impacts on trypanosome infections given the fact that seasonal variations could influence the activity of tsetse flies. Moreover, entomological data on the population dynamics and nutritional behavior of tsetse flies as well as trypanosome infection rates in these flies have not been taken into consideration. Such data could yield additional value to understand the transmission dynamics of trypanosomes between tsetse and vertebrate hosts.

## Conclusion

This study showed that pigs, dogs, sheep and goats are carriers of natural infections of *T. b. gambiense* in Chadian HAT foci. These animals can be considered as potential reservoir hosts of *T. b. gambiense* in Chad. The identification of *T. b. gambiense* in all animal species of all HAT foci suggests that these animals should be considered in the design of control strategies for HAT. As these animals could play a role in the maintenance and resurgence of HAT, further investigations aiming to decrypt their role in each epidemiological setting are important to achieve the WHO goal of zero transmission to humans by 2030.

## Funding

This study was funded through a fellowship offered by the *Organisation de Coordination pour la lutte contre les Endémies en Afrique Centrale* (OCEAC), based on the financial cooperation between the CEMAC and the German Federal Ministry for Economic Cooperation and Development (BMZ) and administered by the Kreditanstalt für Wiederaufbau (KfW)

## Competing interests

The authors declare that they have no conflict of interest.

## Supplementary material

Supplementary material is available at https://www.parasite-journal.org/10.1051/parasite/2020061/olm Table S1 Trypanosome infections according to villages of each HAT focus
