# Supplementary material for: Molecular identification of Trypanosoma brucei gambiense in naturally infected pigs, dogs and small ruminants confirms domestic animals as potential reservoirs for sleeping sickness in Chad
Source: Parasite. 2020 Nov 18;27:63. doi: 10.1051/parasite/2020061 (PMC7673351; doi:10.1051/parasite/2020061)
Supplement: Table S1 — Trypanosome infections according to villages of each HAT focus [file parasite-27-63-s1.pdf]

Table S1: Trypanosome infections according to villages of each HAT focus

HAT foci	Villages	HAT Cases	NE	RDT*	T+	PCR results	
						TB^+	TBG^+
Mandoul	Bembaitada	No	15	6	1	4	1
	Bekolo	No	8	0	0	0	0
	BNK	No	6	2	0	1	0
	Kokoumati II	Yes	8	3	2	2	1
	Kobitoye	Yes	10	3	1	4	2
	Koumouabé	No	12	5	2	1	1
	Ngonbé	No	5	0	0	0	0
	Kokoumati I	Yes	9	0	1	3	2
	Bekonon	No	7	2	0	2	0
	Beyama II	No	5	0	1	0	0
	Bemadja	No	7	1	0	2	0
	Ndosadana	No	6	0	0	0	0
	Koumaingar	No	7	2	0	0	0
	Betoyo	No	4	0	1	0	0
	Missimadji	No	5	1	0	2	0
	Sanodjo	No	8	3	1	3	1
	Kouhsamadja	No	8	1	0	0	0
	Sanodjo Koh	No	5	1	0	2	0
	Berayan	No	9	0	0	1	1
	Beidé II	No	4	0	0	0	0
	Takawa	No	6	0	1	1	0
	Beda	No	5	0	0	0	0
	Ndohsako	Yes	9	4	2	4	1
	Palkoyo	Yes	10	5	2	3	1
	Ndosalte	No	6	1	1	1	0
	Sananga	No	7	0	0	0	0
	Djaraïbé	No	5	1	0	0	0
	Dankou	No	4	0	0	0	0
	Betel	No	6	1	0	0	0
	Jerusalem I	No	3	0	0	1	0
	Ndoayo	No	7	0	1	0	0
	Danmadja	No	14	3	2	3	1
	Benadja	No	5	1	0	0	0
	Bessakoian	No	4	0	0	0	0
	Donditi	No	3	0	0	0	0
	Dedaye I	No	6	1	0	0	0
	Bekili	No	4	0	0	0	0
	Jerusalem II	No	5	1	1	1	0
	Bedoné	No	5	1	0	2	0
	Jerusalem III	No	6	2	0	0	0
Subtotal 1	41		268	51	20	43	12
	Kobdjogué I	Yes	14	5	2	8	1

Maro	Mbimbagué	No	5	1	0	0	0
	Ferme Taguina	No	9	2	1	6	1
	Mobiri	No	5	0	1	2	0
	Sandana	No	7	0	1	0	0
	Kobdjogué II	No	10	2	1	5	0
	Bekion	No	5	1	0	2	0
	Koukourouza	No	7	2	1	3	1
	Dangala	No	4	0	0	0	0
	Sanodjo	No	9	0	1	1	0
	Kion Ngala	No	5	1	0	0	0
	Doro	No	6	1	1	2	0
	Morko	No	4	1	0	0	0
	Ngon Molo	No	5	0	0	0	0
	Ferme Mbaye	No	8	2	1	2	0
	Kaounodjo	No	11	4	1	4	0
	Mouri	No	6	0	1	1	0
	Baguirgué I	No	8	3	2	0	0
	Baguirgué II	No	5	1	0	0	0
	Irnan	No	11	3	1	4	0
	Gourourou	Yes	16	5	2	9	1
	Kira	No	4	1	0	0	0
	Ngon kira	No	6	0	0	0	0
	Sanodjo II	No	8	2	1	0	0
	Morkon II	No	6	0	0	0	0
	Guirkyon	No	10	2	1	0	
	Maikava	No	12	3	1	0	0
	Ndjinaba	No	5	0	0	0	0
	Bekanan	No	6	1	1	1	0
	Ngakorio	No	9	0	0	0	0
	Molo	No	6	1	1	1	0
Subtotal 2	31		232	44	22	51	4
Moissala	Gonhongon	No	8	2	2	5	0
	Lapia	Yes	9	3	2	7	1
	Maikolo	No	4	0	0	0	0
	Moundou	No	4	1	1	3	0
	Dororo	No	8	1	1	5	0
	Effort	No	3	2	0	1	0
	Samadja	No	6	3	0	0	0
	Kougarte	No	5	1	1	3	0
	Kadkou I	No	4	1	0	2	0
	Dodjite	No	3	0	0	0	0
	Ndoyo	No	3	0	0	0	0
	Paris Dakar	No	4	0	1	2	0
	Betoyo	No	3	0	0	0	0
	Bepan II	No	5	3	0	3	0
	Kokoyote	No	4	1	0	2	0
	Berigui	No	3	0	0	0	0
	Sananga	No	3	1	0	0	0

	Bekodo	No	4	1	1	2	0
	Ngokan II	No	3	0	0	1	0
	Mosbé	No	4	0	0	0	0
	Doh Mbanga	No	3	0	0	0	0
	Takaouti	No	3	1	1	1	0
	Kadkou II	No	5	2	1	2	0
	Béka	No	3	0	0	0	0
	Sahoyo	No	3	0	0	0	0
	Bepili	No	7	2	1	4	0
	Ndilbé	No	3	0	0	0	0
	Bendogo	No	3	0	0	0	0
	Dokaouti	No	5	0	1	2	0
	Koudoti	No	4	0	0	0	0
	Brakaba I	No	4	2	0	0	0
	Brakaba II	No	3	1	1	2	0
	Beguina	No	2	0	0	0	0
	Bendanga	No	3	0	0	0	0
	Nanmadja	Yes	7	3	2	5	1
	Kokoumati	No	4	1	0	1	0
	Koubraza	No	4	1	1	2	0
	Kana	No	3	0	0	0	0
	Degaulle	No	4	1	0	0	0
	Mayo	No	3	1	1	1	0
	Maininga	No	3	0	0	0	0
Subtotal 3	41		169	35	18	56	2
**Total**	**113**		**669**	**130**	**60**	**150**	**18**

HAT: Human African Trypanosomiasis; NE: Number of animals examined; RDT: Rapid diagnosis test; T+: trypanosome infections revealed by Capillary tube centrifugation; TB+: trypanosomes of the subgenus *Trypanozoon*: TBG: *Trypanosoma brucei gambiense.*
